# Relative Validity and Reliability of the Remind App as an Image-Based Method to Assess Dietary Intake and Meal Timing in Young Adults

**DOI:** 10.3390/nu15081824

**Published:** 2023-04-10

**Authors:** Catalina Ramírez-Contreras, Andreu Farran-Codina, María Fernanda Zerón-Rugerio, Maria Izquierdo-Pulido

**Affiliations:** 1 Department of Nutrition, Food Science and Gastronomy, Food Science Torribera Campus, University of Barcelona, 08921 Barcelona, Spain; 2 INSA-UB, Nutrition and Food Safety Research Institute, University of Barcelona, 08921 Barcelona, Spain; 3 Department of Fundamental and Medical-Surgical Nursing, Faculty of Medicine and Health Sciences, University of Barcelona, 08907 Barcelona, Spain

**Keywords:** dietary intake assessment, meal timing, food record, image-based dietary records, validation

## Abstract

Image-based dietary records have been validated as tools to evaluate dietary intake. However, to determine meal timing, previous studies have relied primarily on image-based smartphone applications without validation. Noteworthy, the validation process is necessary to determine how accurately a test method measures meal timing compared with a reference method over the same time period. Thus, we aimed to assess the relative validity and reliability of the Remind^®^ app as an image-based method to assess dietary intake and meal timing. For this purpose, 71 young adults (aged 20–33 years, 81.7% women) were recruited for a 3-day cross-sectional study, where they completed a 3-day image-based record using the Remind app (test method) and a 3-day handwritten food record (reference method). The relative validity of the test method versus the reference method was assessed using multiple tests including Bland–Altman, % difference, paired *t*-test/Wilcoxon signed-rank test, Pearson/Spearman correlation coefficients, and cross-classification. We also evaluated the reliability of the test method using an intra-class correlation (ICC) coefficient. The results showed that, compared to the reference method, the relative validity of the test method was good for assessing energy and macronutrient intake, as well as meal timing. Meanwhile, the relative validity of the test method to assess micronutrient intake was poor (*p* < 0.05) for some micronutrients (iron, phosphorus, potassium, zinc, vitamins B1, B2, B3, B6, C, and E, and folates) and some food groups (cereals and grains, legumes, tubers, oils, and fats). Regarding the reliability of an image-based method to assess dietary intake and meal timing, results ranged from moderate to excellent (ICC 95% confidence interval [95% CI]: 0.50–1.00) for all nutrients, food groups (except oils and fats, which had low to moderate reliability), and meal timings. Thus, the results obtained in this study provide evidence of the relative validity and reliability of image-based methods to assess dietary intake (energy, macronutrients, and most food groups) and meal timing. These results open up a new framework for chrononutrition, as these methods improve the quality of the data collected and also reduce the burden on users to accurately estimate portion size and the timing of meals.

## 1. Introduction

The future of chrononutrition relies on what we can learn about individual behavior, which requires an accurate assessment of what people eat and drink, as well as when and how often they consume any type of food or beverages [1,2]. The latter are also known as temporal eating patterns [2]. Commonly used methods to assess temporal eating patterns include pen-and-paper tools such as food records and 24 h dietary recalls [3,4,5]. However, these tools can be complex and burdensome for the users [1], especially considering that they require the ability to estimate and remember portion sizes as well as the preparations of consumed foods. In addition, the intake of snacks is commonly associated with social interactions and, as such, logging food intake and timing can also be a burden in free-living conditions [2]. However, beyond the load for the users, the latter is also a large source of bias in dietary assessment for researchers [1,3].

Fortunately, with the development of technology, other approaches have emerged, such as the use of digital food photography, to help users to document food and beverage intake [1,6,7]. Along with this, the use of digital food photography has prompted an improvement in dietary assessment [6], reducing reliance on the participant’s memory, providing the researcher with direct visual documentation of what is eaten, and reducing underreporting [1,2,8]. Currently, there are two main approaches in which digital food photography can be used: image-assisted dietary records and image-based dietary records [9,10]. On the one hand, image-assisted methods consist of capturing images with handheld devices or wearable cameras, as an aid to estimate portion sizes or to remember the foods consumed [8]. On the other hand, image-based methods aim to capture all eating occasions through images as the primary record of dietary intake and thus follow the methodology of food recording [8]. Consequently, the use of image-based dietary records over traditional pen-and-paper dietary records has been very well received by users and researchers [7,8,11,12].

Image-based dietary records are currently delivered by users via smartphone applications, which have been validated as tools to evaluate dietary intake [7,13,14]. However, to determine meal timing, previous studies have relied primarily on image-based smartphone applications without validation [2,15]. Importantly, the validation process is necessary to determine how accurately a test method measures meal timing compared with a reference method over the same time period (known as relative validity) [16]. Furthermore, the validation process is necessary to identify the magnitude and direction of measurement error, the potential causes of measurement error, and how these errors can be minimized or accounted for in the analyses [16]. Of note, a recent validation study by Giogia et al. [17] showed significant differences in the timing of most meals, when reported through recall-based survey questions versus paper-based food records. Specifically, the authors showed a significant delay in meal timings when reported through recall-based surveys, compared to those reported through food records [17]. These results highlight the relevance of evaluating the relative validity of image-based dietary records to assess meal timing, versus a reference method. 

Taking the above into account, our aim was to assess the relative validity and reliability of the Remind app [18] as an image-based method to jointly assess dietary intake and meal timing versus food recording. Note that the Remind app is a real-time mobile application that allows for file and photo sharing, as well as immediate feedback from respondents [18]. The latter is relevant considering that daily life and work styles have been characterized by instantaneous communication that is increasingly accessible via digital technology [19]. Furthermore, the COVID-19 pandemic has accelerated the use of telemedicine, making the use of emerging technologies in clinical practice more likely [1]. From this perspective, we emphasize the need for new validated applications to jointly assess dietary intake and meal timing. 

## 2. Materials and Methods

### 2.1. Participants and Study Design

Young adults (aged 20–35 years) were recruited for a 3-day cross-sectional study among undergraduate students at the University of Barcelona (Barcelona, Spain). Recruitment consisted of an informative talk, explaining the details to the volunteers about the research, and inviting them to take part in the study. Exclusion criteria consisted of not owning or having access to a smartphone capable of downloading the Remind app and/or unwillingness to participate in the study. Based on these criteria, a total of 78 subjects were included in the study, and all of whom gave their written informed consent. We further excluded 7 subjects with missing information in their food records (e.g., serving sizes, method of preparation) which resulted in a final analytical sample of 71 participants. All study procedures were conducted according to the general recommendations of the Declaration of Helsinki and were approved by the Ethics Committee of the University of Barcelona (IRB00003099). 

### 2.2. Anthropometric Measurements

Weight was measured using a body composition analyzer (InBody 720, Biospace, Seoul, Korea), with the subjects wearing light clothing and without shoes, to the nearest 0.1 kg. Height was determined using a fixed wall stadiometer (Seca 217, Seca, Hamburg, Germany) to the nearest 0.1 cm. Body mass index (BMI) was calculated as weight (kg) divided by height squared (m). 

### 2.3. Dietary Intake and Meal Timing Assessment Methods

During the study period, all participants were asked to complete a handwritten 3-day food record (reference method) and a 3-day image-based dietary record using the Remind app (test method) [18]. Participants were asked to complete both records within one week, but on the same days and including two weekdays and one weekend day. In addition, a registered dietitian instructed participants to record the type of food or beverage (including alcoholic beverages) with the brand, if possible, the method of preparation, the serving size (in grams or household measurements), and the location of the meal (e.g., home or restaurant). In addition, participants were required to report meal times in both methods during the study period. This allowed us to evaluate the time and frequency in which each food or beverage was consumed. 

**Figure 1:**
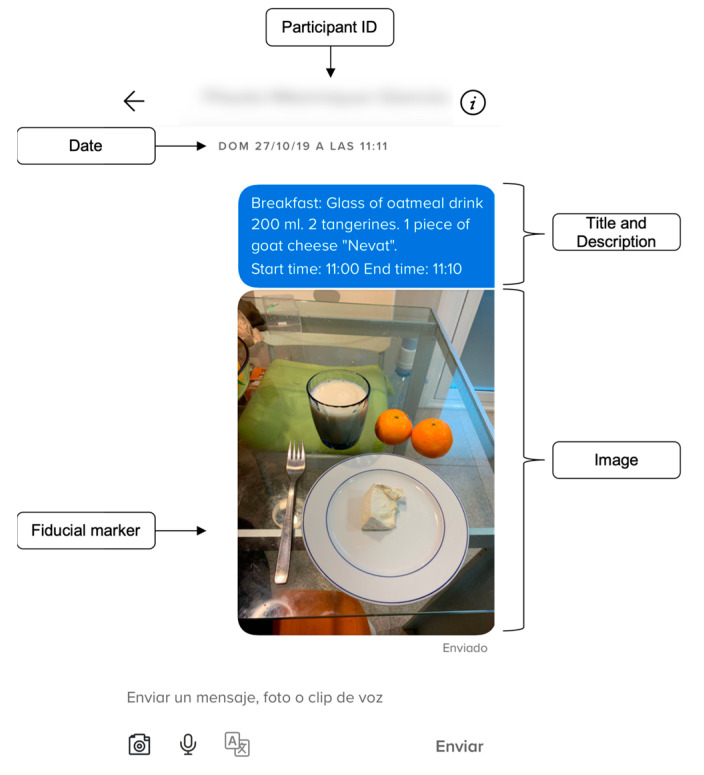
Example of an eating occasion recorded in the Remind app, including supporting text description, fiducial marker, date, and title of record.

For the image-based dietary record, participants were asked to download the Remind app into their mobile phones. It should be noted that the Remind^®^ app complies with the requirements of the EU General Data Protection Regulation, is free, and is compatible with all mobile operating systems [18]. Additionally, all participants received training on how to use the mobile app and how to take the pictures so that they could accurately reflect food intake (Figure 1). As such, participants were taught to photograph all foods/beverages consumed at a 45° angle using a fiducial marker (a reference object with known dimensions), which could be a pen or cutlery [20]. Participants were also asked to take pictures of second servings and leftovers. In addition, as shown in Figure 1, participants were required to enter a brief written description of what they ate, as well as the time the food was consumed. It is worth noting that the Remind app provides real-time communication to monitor participants’ progress, which can reduce participant burden and improve data quality.

#### 2.3.1. Dietary Intake

Data from handwritten food records and image-based dietary records were processed by a registered dietitian using PCN Pro 1.0 software [21]. To estimate the daily dietary intake for both methods, we standardized the food entries, including selected food portions, according to values provided in the software (i.e., a normal serving of pasta), unless the participant provided the exact quantity of food or beverage. Furthermore, specific brands were not selected unless indicated by the participant. Additionally, we used photographic guides of food portions consumed in Spain [22,23] to help to quantify the foods included in the image-based dietary records. The latter allowed us to estimate the average daily energy (kcal/day), macronutrient (g/day), and micronutrient (mg/day or μg/day) intakes for both methods. In addition, we estimated the average daily intake (g/day) of the following food groups:

- Fruits: fresh fruits, canned fruits, and dried fruits.
- Vegetables: leaf, flower, or stem vegetables, root vegetables, bulbs, and mushrooms.
- Cereals and grains: cereals, grains and flour, pasta, baked goods, cookies, pastries, and breakfast cereals.
- Legumes: legumes, dry legumes, legume flour, and derivatives.
- Tubers: potatoes and other starchy tubers.
- Milk and dairy products: milk and milkshakes, yogurt and fermented milk, dairy desserts, fresh cheese, aged cheese, processed cheese, and milk ice cream or similar.
- Meats: pork, veal, lamb, beef, rabbit, poultry, viscera, and raw, raw cured, and heat-treated sausages.
- Eggs: chicken eggs and other eggs from other birds.
- Fish: cod, hake, salmon, tuna, sole, monkfish, mackerel, sardines, etc.
- Oils and fats: olive oil, sunflower oil, coconut oil, lard, butter, and margarine.
- Non-alcoholic drinks: coffee, cocoa, infused beverages, mineral water, soda, juices, and packaged nectars.

#### 2.3.2. Meal Timing 

For each meal, participants were asked to record the time in which they started eating on handwritten food records, as well as on the image-based food records. Note that in both cases, the participants recorded meal times based on the time of their cellphones. Subsequently, meals were classified as breakfast, lunch, dinner, or mid-morning and mid-afternoon snacks, based on the designation that each participant indicated. We then calculated the average meal timing in which breakfast, lunch, dinner, and mid-morning and mid-afternoon snacks were consumed. 

### 2.4. Validation Process of Remind as an Image-Based Method to Assess Dietary Intake and Meal Timing 

#### 2.4.1. Relative Validity

This parameter was determined by comparing a test method (Remind app) to a reference method (3-day handwritten food records), where the reference method had a higher degree of demonstrated validity, although it was not an exact measure of the underlying concept [24]. For this purpose, we applied the methodology proposed by Lombard et al. [16], where a combination of 5 statistical tests (Bland–Altman, % difference, paired *t*-test/Wilcoxon signed-rank test, Pearson/Spearman correlation coefficients, and cross-classification) is used to test different facets of validity such as agreement, association, or bias, either at the group or individual levels [16].

##### Agreement at Group Level

To test group-level agreement, we first used the Bland–Altman test, which reflects the presence, direction, and extent of bias, as well as the limits of agreement [16,25]. The latter was assessed by plotting the mean difference (y-axis) and the mean intakes (x-axis) between both methods and for each subject, to illustrate the magnitude of disagreement and to identify outliers and trends in bias [16,25]. In this case, the mean difference and mean intakes were calculated as follows:

Mean difference = (test measure − reference measure)

Mean intake = [(test measure + reference measure)/2]

Then, the upper and lower limits of agreement were calculated as follows:

Lower limit of agreement (LLA) = mean difference − 1.96 standard deviations

Upper limit of agreement (ULA) = mean difference + 1.96 standard deviations

Note that about 95% of the recordings should be between the LLA and the ULA. 

In addition, Bland–Altman Spearman correlation coefficients between mean difference and mean intake were calculated to reflect the presence of proportional bias as well as its direction [16]. Then, according to the *p*-value, the outcomes were classified as “good” (*p* > 0.05) or “poor” (*p* ≤ 0.05). In this case, poor outcomes would reflect proportional bias [16]. 

We also calculated the difference (in percentage) between the reference and the test measure. The latter reflects the size and direction of the error at the group level [16]. The % difference was calculated for the total sample as follows:

% Difference = [(Test measure − reference measure)/reference measure] × 100

According to the % difference, the outcomes were classified as “good” (0.0–10.9%), “acceptable” (11.0–20.0%), or “poor” (>20.0%) [16].

Subsequently, we used the paired *t*-test (parametric) or the Wilcoxon signed-rank test (non-parametric) to evaluate the agreement between the test and reference methods [26,27]. Then, according to the *p*-value, the outcomes were classified as “good” (*p* > 0.05) or “poor” (*p* ≤ 0.05) [27].

##### Agreement at Individual Level 

First, we measured the strength of the association between the test and the reference method [27]. This parameter was assessed based on the data distribution, using Pearson’s (parametric) or Spearman’s (non-parametric) correlation coefficients. Then, according to the correlation coefficient (r/Rho), the outcomes were classified as “good” (≥0.50), “acceptable” (0.20–0.49), or “poor” (<0.20) [28].

We also used the cross-classification method, which provides an indication of how well the dietary method separates subjects into classes or consumption categories [27]. To do so, we used tertiles and then calculated the percentage of subjects who were correctly classified in the same tertile. Likewise, we estimated the percentage of subjects who were in the opposite category overall [27]. Then, according to the % of subjects who were correctly or grossly classified in each tertile, the outcomes were classified as follows [28]:

- “Good” if ≥50% of the sample was classified in the same tertile and ≤10% in the opposite tertile.
- “Poor” if <50% of the sample was classified in the same tertile and >10% in the opposite tertile.

#### 2.4.2. Reliability

Reliability refers to the extent to which a measurement process gives the same results when repeated under similar circumstances [24]. Therefore, this parameter is defined as the extent to which measurements can be replicated [29]. To evaluate this parameter, we used the intraclass correlation coefficient (ICC) based on a 2-way random-effects model, which is a widely used reliability index that reflects the absolute agreement between measurements of the same quantitative variable, in the same subjects [29]. Note that the appropriate ICC interpretation to evaluate the level of reliability should be based on the 95% confidence interval [95% CI] of the ICC estimate, not the ICC estimate itself [29]. Thus, according to the ICC’s [95% CI], the outcomes were classified as “excellent” (>0.90), “good” (>0.75–0.90), “moderate” (0.50–0.75), or “poor” (<0.50) [29].

### 2.5. Statistical Analyses

Normality was confirmed for all variables using histograms and Q-Q plots. Descriptive characteristics were presented for all participants, including mean ± standard deviation for parametric variables, median (interquartile range) for non-parametric variables, and proportions for categorical variables. Then, for the validation process of the Remind app (test measure) versus the 3-day handwritten food records (reference method), we constructed Bland–Altman plots and calculated Bland–Altman Spearman correlation coefficients to assess any systematic bias between methods. In addition, we calculated the % difference and compared the differences between methods using paired *t*-test/Wilcoxon signed-rank tests to assess agreement at the group level. We then evaluated individual-level agreement using the correlation coefficient (Pearson or Spearman, based on data distribution) and cross-classification. Finally, we calculated the ICC [95% CI] based on a 2-way random-effects model to assess reliability. All analyses were performed using SPSS statistical computer software, version 25.0 (IBM SPSS Statistics, Armonk, NY, USA).

## 3. Results

Briefly, 71 young adults (81.7% women; aged 22.5 ± 2.3 years; BMI 22.3 ± 3.2 kg/m^2^) were included in this study. The mean values of estimated dietary intake as seen via the test and reference methods are shown in Table S1. Overall, the average energy intake was 1485.6 ± 301.4 kcal/day, while the macronutrient intake was as follows: 157.9 ± 34.5 g/day of carbohydrates, 78.4 ± 18.3 g/day of proteins, and 59.4 ± 16.9 g/day of fat. Regarding the timing of food intake, on average, participants had breakfast, lunch, and dinner at 09:27 ± 01:00, 14:11 ± 00:34, and 21:31 ± 00:35, respectively. In addition, the average timings of mid-morning and mid-afternoon snacks were at 11:23 ± 01:05 and 17:57 ± 00:56, respectively.

### 3.1. Relative Validity of Remind App as a Tool to Evaluate Dietary Intake and Meal Timing 

#### 3.1.1. Energy and Nutrient Intake

**Figure 2:**
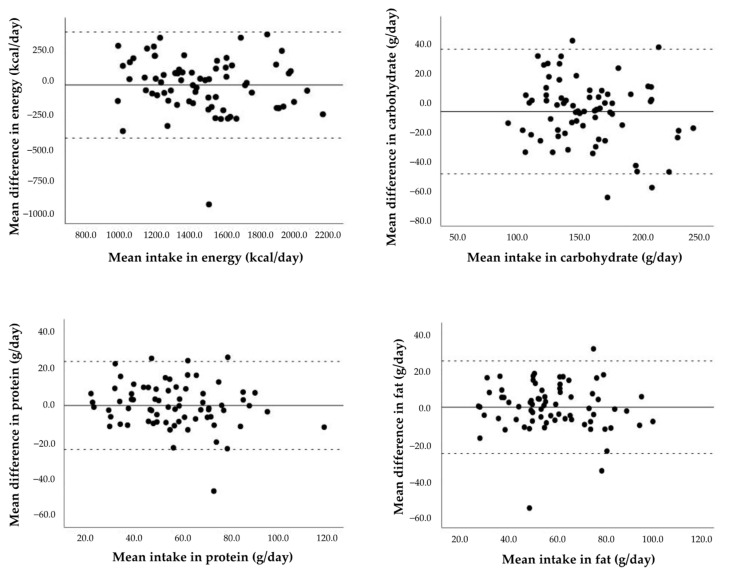
Bland–Altman plots showing the mean difference vs. the mean intake (solid line) between the test (Remind app) and reference (3-day handwritten food records) methods, and the lower and upper limits of agreement (dotted lines) for energy and macronutrient intake.

The agreement at the group level was analyzed using Bland–Altman plots to compare the mean differences in energy and nutrient intakes between the test and the reference method. As shown in Figure 2, most values of energy (kcal/day) and macronutrient intake (carbohydrate (g/day), protein (g/day), and fat intake (g/day)) were within acceptable limits of agreement. Likewise, most of the values regarding micronutrient intake were within acceptable limits of agreement. 

Furthermore, according to the Bland–Altman Spearman correlation coefficients (Table 1), we found good outcomes (*p* > 0.50) for energy, macronutrients, dietary fiber, and most micronutrients. However, the outcomes for iron (*p* = 0.031), vitamin E (*p* = 0.006), and vitamin C (*p* = 0.042) intakes were considered to be poor and thus reflected proportional bias. 

Regarding the % difference (Table 1), our results showed that energy and all nutrient intakes had a difference of <11%, which indicates a good outcome. Likewise, the results from the paired *t*-test/Wilcoxon signed-rank test (Table 1) showed a good outcome for energy, protein, fat, and fiber intakes, as well as calcium, magnesium, phosphorus, and sodium intakes, and vitamins A, D, and B12 intakes. Meanwhile, carbohydrate and the remaining micronutrient (iron, phosphorus, potassium, zinc, vitamins E, B1, B2, B3, B6, C, and folate) intakes showed poor agreement at the group level. 

As for the individual level of agreement between the test and the reference method (Table 1), we observed that the outcomes for the correlation coefficients and cross-classification analyses were good for all items (energy and nutrient intake). 

#### 3.1.2. Food Group Intake

The Bland–Altman plots of the intake of the different food groups showed that most values were within the acceptable limits of agreement (Figure S1). In addition, according to the Bland–Altman Spearman correlation coefficients (Table 2), there was a good outcome for most of the food groups (fruits, vegetables, cereals and grains, legumes, milk and dairy products, meats, eggs, fish, and non-alcoholic drinks), except for tubers (*p* = 0.003) and oils and fats (*p* = 0.028) where the outcome was poor, indicating that there was proportional bias. 

Regarding the % difference, our results revealed that most of the food groups (fruits, vegetables, cereals and grains, tubers, milk and dairy products, meats, and fish) had good agreement at the group level. Meanwhile, eggs and non-alcoholic beverages had an acceptable level of agreement, while legumes and oils and fats showed a poor group-level agreement (Table 2). Likewise, according to the paired *t*-test/Wilcoxon signed-rank test (Table 2), most food groups showed good agreement at the group level, except for cereals and grains and tubers, which showed poor agreement at the group level. 

Concerning the relative validity at the individual level (Table 2), the correlation coefficients and the results of the cross-classification analyses showed that the intakes of all of the food groups exhibited a strong relationship at the individual level. Only oils and fats showed a poor outcome in the cross-classification analysis, with 14% of individuals being misclassified in the opposite tertile. 

#### 3.1.3. Meal Timing 

**Figure 3:**
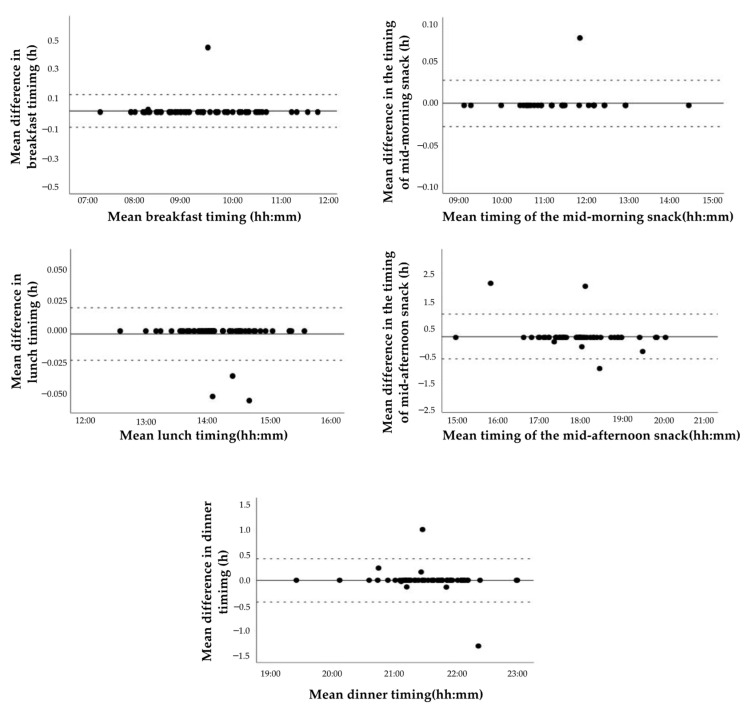
Bland–Altman plots showing mean difference vs. mean timing (solid line) between the test (Remind app) and reference (3-day handwritten food records) methods, and the lower and upper limits of agreement (dotted lines) for breakfast, mid-morning snack, lunch, mid-afternoon snack, and dinner timings.

As shown in Figure 3, the Bland–Altman plots showed that most meal timing values fell within the acceptable limits of agreement. In addition, the results of the Bland–Altman Spearman correlation coefficients, the % difference, and the paired *t*-test/Wilcoxon signed-rank tests revealed that the timing of all meals, reported using the test method, had good agreement at the group level compared to the reference method (Table 3). Similarly, the agreement at the individual level of the test method evaluated using correlation coefficients and cross-classification showed good outcomes for all meal times (Table 3).

### 3.2. Reliability of the Remind App as a Tool to Evaluate Dietary Intake and Meal Timing 

Regarding the reliability of the Remind app (Table 4), we observed that according to the ICC [95% CI], energy intake had moderate to excellent reliability, while carbohydrates, and fats and fatty acids (saturated, monounsaturated, and polyunsaturated) had moderate to good reliability. We also found that the reliability of the test method to assess protein, cholesterol, and dietary fiber intake could be considered as good to excellent. Likewise, its reliability to evaluate several vitamin intakes (A, D, B1, B6, B12, and folates) was good to excellent, while the reliability in assessing magnesium was good and the reliability in assessing other mineral intakes (calcium, iron, phosphorus, potassium, and zinc) and some vitamin intakes (E, B2, B3, and C) was moderate to good.

Among food groups (Table 4), the reliability of the test method to evaluate fish intake was excellent, while for legumes and tubers the reliability was good to excellent. For the other food groups (fruits, vegetables, cereals and grains, milk and dairy products, meats, eggs, and non-alcoholic drinks), the ICC [95% CI] indicated that the Remind app had moderate to good reliability, while for oils and fats the reliability was poor to moderate. 

Finally, our results showed that the reliability of the test method to evaluate the timing of most meals was excellent. Only in the case of the timing of the mid-afternoon snack and dinner was the reliability good to excellent (Table 4). 

## 4. Discussion

Our results showed that, compared to the reference method, the Remind app has good relative validity and moderate to excellent reliability to jointly assess dietary intake (energy, macronutrient, and food groups) and meal timing. To our knowledge, this is the first study to evaluate the relative validity and reliability of a mobile app as a tool to assess meal timing. In this regard, our results suggest that image-based records have good agreement at the individual and group levels for assessing meal timing. The latter is important considering that temporal eating patterns, that is, what and when we eat, are important contributors to health [2,30]. Furthermore, evidence from experimental and epidemiological studies in the field of chrononutrition has shown the importance of meal timing and its implications in obesity and its management [2,31,32,33]. Therefore, validated tools, such as ours, are needed to capture the temporal components of energy and nutrient intake.

Among other significant findings, our results showed that the Remind app had good reliability for assessing the average daily consumption (g/day) of fruits, vegetables, milk and dairy products, meats, eggs, fish, and non-alcoholic beverages. This is in agreement with Matthiessen et al. [13] who evaluated the relative validity of image-based records to assess food group intake. Only in the case of cereals and grains, legumes, tubers, oils and fats were the outcomes suboptimal. In this regard, Boushey et al. [8] postulated that these differences may be due to the fact that a photograph can provide more information than paper-based food records. In our experience, this could be the case for oils and fats, where some participants did not report the use of oil for cooking or salad dressing in paper-and-pen food records, while in image-based records the presence of oil could be clearly seen. This observation is in line with another study, which also indicates that the food group “oils and fats” is commonly misclassified and thus reflects poor agreement at the individual and group levels [34]. 

Regarding the differences at the group level in the intake of legumes, cereals, and tubers, we hypothesized that the discrepancies could lie in the way in which users and experts (in our study, a registered dietitian) estimate the size of the portions consumed [1,3]. Note that user estimation of portion size is a well-established limitation of pen-and-paper food records [1], and so it is plausible that image-based records may provide a better estimate of portion sizes. The latter could also be in line with Boushey’s observation, noting that a photograph can provide more information than paper-based food records. However, it should be taken into account that this type of study investigates the “relative validity” of one method with respect to another, which implies that neither has the absolute truth [7,16].

Among other significant findings, our results showed that the use of the Remind app as an image-based method has good relative validity and a moderate to excellent reliability to assess energy and macronutrient intakes. Similar results were found in other validation studies of image-based methods, where data from energy and macronutrient intakes were within the limits of agreement [14,34]. Furthermore, the results of a systematic review and meta-analysis showed that when used as a primary dietary record, image-based dietary assessments can provide valid results for assessing energy and macronutrient intakes, as with traditional methods (i.e., 24 h dietary recall and estimated/weighted food records) [9]. 

Although, as with any other method to assess dietary intake [7], image-based records provided through the Remind app had their limitations with regard to micronutrient assessment. As such, compared to the reference method, the Remind app showed some differences at the group level when assessing the intake of iron, potassium, zinc, vitamins E, B1, B2, B3, B6, and C, and folates. These results are partially consistent with other validation studies that also found low agreement between image-based records and food records to assess the intake of iron, zinc, and folates [7,35]. 

Despite our findings, some caution needs to be taken when interpreting our results. First, this study included a relative validation; therefore, it is not possible to conclude that one method is closer to the “true dietary intake” than the other [7]. Second, previous research has shown that three days may be adequate for establishing the mean energy intakes of groups; however, it may not be a long enough duration to accurately measure micronutrient intake [36]. Third, our final sample included 71 young adults, which while similar to other studies that have assessed the relative validity of image-based methods to assess dietary intake [14,37,38], may not be representative of the entire population. Fourth, we also recognize that our sample of young adults may not be representative of the entire population with respect to technology use. Therefore, future studies are needed to assess the relative validity of the Remind app for assessing dietary intake and meal timing in other populations. Nonetheless, our study had several strengths, beginning with the fact that we used a combination of five different tests to evaluate the relative validity and reliability. These, according to Lombard et al. [16], reflect different facets of validity such as agreement, association, or bias at the group or individual level. Second, our study was focused on the validation of the temporal aspects of food intake; so, we evaluated the relative validity of the app to jointly assess dietary intake (energy, nutrients, and food groups) and meal timing in free-living conditions. Third, in the image-based dietary records, participants provided written information in addition to the photograph which may have improved the accuracy of estimates [37]. Fourth, a registered dietitian performed the dietary data entry and analysis. Finally, the Remind app allowed us to access real-time communication to monitor participant progress, potentially reducing the participant burden and improving data quality.

## 5. Conclusions

In summary, our results provide evidence of the relative validity and reliability of an image-based method to assess the temporal aspects of food intake. As such, the Remind app has good relative validity and moderate to excellent reliability to jointly assess dietary intake (energy, macronutrients, and most food groups) and meal timing. The latter opens a new framework for chrononutrition, as these image-based methods improve the quality of data collected and also reduce the burden on users to accurately estimate portion size and meal timing. Furthermore, considering that our current lifestyles are characterized by technology and instant communication, the use of novel technologies to assess dietary intake and meal timing in clinical and epidemiological settings is necessary. 

## Figures and Tables

**Table 1:** Summary of statistical test outcomes and interpretation of energy and nutrient intakes to assess the relative validity of Remind app.

	Agreement at Group Level			Agreement at Individual Level		
	Bland–Altman Spearman Correlation Coefficient, *p*-Value	Difference, %	Paired *t*-Test/Wilcoxon Signed-Rank Test, *p*-Value	Correlation Coefficient, r or Rho	Cross-Classification	
					Same Tertile, %	Opposite Tertile, %
Energy, kcal/day	0.099 ●	−0.7 ●	0.205 ●	0.798 ●	64.8 ●	1.4 ●
Macronutrients						
Carbohydrate						
g/day	0.200 ●	−2.6 ●	0.017 ○	0.835 ●	73.3 ●	1.4 ●
%TEI	0.238 ●	−1.4 ●	0.150 ●	0.742 ●	63.3 ●	7.0 ●
Protein						
g/day	0.212 ●	1.0 ●	0.680 ●	0.812 ●	67.6 ●	1.4 ●
%TEI	0.618 ●	2.0 ●	0.243 ●	0.859 ●	69.0 ●	1.4 ●
Fat						
g/day	0.455 ●	2.3 ●	0.834 ●	0.752 ●	64.8 ●	1.4 ●
%TEI	0.586 ●	2.2 ●	0.311 ●	0.739 ●	67.6 ●	1.4 ●
Saturated fat, g/day	0.801 ●	1.6 ●	0.984 ●	0.840 ●	67.7 ●	0.0 ●
Monounsaturated fat, g/day	0.104 ●	2.8 ●	0.286 ●	0.664 ●	57.7 ●	4.2 ●
Polyunsaturated fat, g/day	0.932 ●	3.6 ●	0.986 ●	0.782 ●	66.1 ●	2.8 ●
Cholesterol, mg/day	0.616 ●	5.3 ●	0.687 ●	0.856 ●	70.4 ●	0.0 ●
Dietary fiber, g/day	0.371 ●	0.3 ●	0.404 ●	0.906 ●	77.5 ●	1.4 ●
Micronutrients						
Calcium, mg/day	0.916 ●	−2.3 ●	0.080 ●	0.807 ●	78.9 ●	0.0 ●
Iron, mg/day	0.031 ○	−2.2 ●	0.035 ○	0.815 ●	67.6 ●	2.8 ●
Magnesium, mg/day	0.052 ●	−0.6 ●	0.207 ●	0.856 ●	67.7 ●	1.4 ●
Phosphorus, mg/day	0.202 ●	−2.0 ●	0.047 ●	0.845 ●	50.7 ●	1.4 ●
Potassium, mg/day	0.116 ●	−5.5 ●	<0.001 ○	0.861 ●	67.6 ●	1.4 ●
Zinc, mg/day	0.068 ●	−2.4 ●	0.021 ○	0.835 ●	57.7 ●	1.4 ●
Vitamin A, μg/day	0.100 ●	2.7 ●	0.460 ^a^ ●	0.865 ^b^ ●	74.6 ●	2.8 ●
Vitamin D, μg/day	0.760 ●	0.4 ●	0.351 ^a^ ●	0.858 ^b^ ●	70.5 ●	1.4 ●
Vitamin E, mg/day	0.006 ○	−1.3 ●	0.040 ○	0.826 ●	66.2 ●	1.4 ●
Vitamin B1, mg/day	0.133 ●	−3.5 ●	0.009 ○	0.868 ●	70.4 ●	0.0 ●
Vitamin B2, mg/day	0.560 ●	−2.5 ●	0.029 ○	0.827 ●	71.9 ●	0.0 ●
Vitamin B3, mg/day	0.833 ●	−5.2 ●	0.015 ○	0.805 ●	71.8 ●	2.8 ●
Vitamin B6, mg/day	0.854 ●	−3.6 ●	0.019 ○	0.856 ●	73.2 ●	2.8 ●
Folates, μg/day	0.301 ●	−5.0 ●	0.006 ○	0.868 ●	66.2 ●	0.0 ●
Vitamin B12, μg/day	0.612 ●	−2.7 ●	0.103 ^a^ ●	0.902 ^b^ ●	73.3 ●	0.0 ●
Vitamin C, mg/day	0.042 ○	−9.7 ●	<0.001 ○	0.861 ●	69.0 ●	0.0 ●

TEI, total energy intake. Outcomes: ● good; ○ poor. ^a^ Wilcoxon signed-rank test. ^b^ Spearman correlation coefficient; Spearman correlation coefficient of Bland–Altman (*p*-value): good: *p* > 0.05; poor: *p* ≤ 0.05. Difference (%): good: 0.0–10.9%; acceptable: 11.0–20.0%; poor: >20.0%. Paired *t*-test/Wilcoxon signed-rank test (*p*-value): good: *p* > 0.05; poor: *p* ≤ 0.05. Correlation coefficient (r or Rho): good: ≥0.50; acceptable: 0.20–0.49; poor: <0.20. Cross-classification (same tertile, %): good ≥ 50%; poor: <50%. Cross-classification (opposite tertile, %): good ≤ 10%; poor > 10%.

**Table 2:** Summary of statistical test outcomes and interpretation of food group intakes to assess the relative validity of Remind app.

		Agreement at Group Level			Agreement at Individual Level		
		Bland–Altman Spearman Correlation Coefficient, *p*-Value	Difference, %	Paired *t*-Test/Wilcoxon Signed-Rank Test, *p*-Value	Correlation Coefficient, r or Rho	Cross-Classification	
						Same Tertile, %	Opposite Tertile, %
Fruits, g/day	0.334 ●	−1.4 ●	0.315 ^a^ ●		0.926 ^b^ ●	77.5 ●	0.0 ●
Vegetables, g/day	0.155 ●	−3.8 ●	0.122 ^a^ ●		0.856 ^b^ ●	67.6 ●	1.4 ●
Cereals and grains, g/day	0.327 ●	9.7 ●	0.008 ○		0.799 ●	62.0 ●	2.8 ●
Legumes, g/day	0.494 ●	20.6 ○	0.510 ^a^ ●		0.923 ^b^ ●	80.3 ●	0.0 ●
Tubers, g/day	0.003 ○	−6.9 ●	0.005 ^a^ ○		0.928 ^b^ ●	83.1 ●	0.0 ●
Milk and dairy products, g/day	0.685 ●	2.2 ●	0.081 ●		0.799 ●	63.4 ●	1.4 ●
Meats, g/day	0.135 ●	1.0 ●	0.132 ^a^ ●		0.846 ^b^ ●	80.3 ●	0.0 ●
Eggs, g/day	0.506 ●	11.8 ◒	0.589 ^a^ ●		0.830 ^b^ ●	73.2 ●	1.4 ●
Fish, g/day	0.812 ●	−5.4 ●	0.223 ^a^ ●		0.943 ^b^ ●	88.8 ●	0.0 ●
Oils and fats, g/day	0.028 ○	31.8 ○	0.877 ^a^ ●		0.518 ^b^ ●	52.1 ●	14.0 ○
Non-alcoholic drinks, g/day	0.343 ●	−20.0 ◒	0.312 ^a^ ●		0.847 ^b^ ●	74.7 ●	1.4 ●

Outcome: ● good; ◒acceptable; ○ poor. ^a^ Wilcoxon signed-rank test. ^b^ Spearman correlation coefficient. Spearman correlation coefficient of Bland–Altman (*p*-value): good: *p* > 0.05; poor: *p* ≤ 0.05. Difference (%): good: 0.0–10.9%; acceptable: 11.0–20.0%; poor: >20.0%. Paired *t*-test/Wilcoxon signed-rank test (*p*-value): good: *p* > 0.05; poor: *p* ≤ 0.05. Correlation coefficient (r or Rho): good: ≥0.50; acceptable: 0.20–0.49; poor: <0.20. Cross-classification (same tertile, %): good ≥ 50%; poor: <50%. Cross-classification (opposite tertile, %): good ≤ 10%; poor > 10%.

**Table 3:** Summary of statistical test outcomes and interpretation of meal timings to assess the relative validity of Remind app.

	Agreement at Group Level			Agreement at Individual Level		
	Bland–Altman Spearman Correlation Coefficient, *p*-Value	Difference, %	Paired *t*-Test/Wilcoxon Signed-Rank Test, *p*-Value	Correlation coefficient, r or Rho	Cross-Classification	
					Same Tertile, %	Opposite Tertile, %
Breakfast, hh:mm	0.500 ●	0.1 ●	0.304 ●	0.998 ●	100.0 ●	0.0 ●
Mid-morning snack, hh:mm	0.490 ●	0.0 ●	0.325 ●	1.000 ●	97.0 ●	0.0 ●
Lunch, hh:mm	0.350 ●	−0.0 ●	0.088 ●	1.000 ●	96.7 ●	0.0 ●
Mid-afternoon snack, hh:mm	0.228 ●	0.2 ●	0.580 ●	0.907 ●	94.4 ●	1.9 ●
Dinner, hh:mm	0.415 ●	−0.0 ●	0.908 ●	0.933 ●	93.4 ●	1.7 ●

Outcome: ● good. Spearman correlation coefficient of Bland–Altman (*p*-value): good: *p* > 0.05; poor: *p* ≤ 0.05. Difference (%): good: 0.0–10.9%; acceptable: 11.0–20.0%; poor: >20.0%. Paired *t*-test/Wilcoxon signed-rank test (*p*-value): good: *p* > 0.05; poor: *p* ≤ 0.05. Correlation coefficient (r or Rho): good: ≥0.50; acceptable: 0.20–0.49; poor: <0.20. Cross-classification (same tertile, %): good ≥ 50%; poor: <50%. Cross-classification (opposite tertile, %): good ≤ 10%; poor > 10%.

**Table 4:** Summary of intra-class correlation coefficient (ICC) to assess the reliability of Remind app.

Dietary Intake	ICC [95% CI]	Interpretation ^1^
Energy, kcal/day	0.793 [0.688, 0.928]	Moderate to excellent
Macronutrients		
Carbohydrate		
g/day	0.820 [0.719, 0.886]	Moderate to good
%TEI	0.733 [0.605, 0.825]	Moderate to good
Protein		
g/day	0.809 [0.711, 0.877]	Moderate to good
%TEI	0.858 [0.782, 0.909]	Good to excellent
Fat		
g/day	0.754 [0.632, 0.839]	Moderate to good
%TEI	0.739 [0.612, 0.829]	Moderate to good
Saturated fat, g/day	0.841 [0.757, 0.898]	Good
Monounsaturated fat, g/day	0.652 [0.496, 0.767]	Moderate to good
Polyunsaturated fat, g/day	0.785 [0.675, 0.860]	Moderate to good
Cholesterol, mg/day	0.856 [0.778, 0.907]	Good to excellent
Dietary fiber, g/day	0.906 [0.853, 0.940]	Good to excellent
Micronutrients		
Calcium, mg/day	0.802 [0.700, 0.872]	Moderate to good
Iron, mg/day	0.803 [0.698, 0.873]	Moderate to good
Magnesium, mg/day	0.851 [0.772, 0.904]	Good
Phosphorus, mg/day	0.837 [0.749, 0.896]	Moderate to good
Potassium, mg/day	0.836 [0.710, 0.904]	Moderate to good
Zinc, mg/day	0.821 [0.722, 0.886]	Moderate to good
Vitamin A, μg/day	0.880 [0.814, 0.923]	Good to excellent
Vitamin D, μg/day	0.896 [0.839, 0.934]	Good to excellent
Vitamin E, mg/day	0.805 [0.703, 0.875]	Moderate to good
Vitamin B1, mg/day	0.855 [0.767, 0.909]	Good to excellent
Vitamin B2, mg/day	0.815 [0.716, 0.882]	Moderate to good
Vitamin B3, mg/day	0.793 [0.680, 0.868]	Moderate to good
Vitamin B6, mg/day	0.848 [0.761, 0.904]	Good
Folates, μg/day	0.855 [0.765, 0.910]	Good to excellent
Vitamin B12, μg/day	0.917 [0.868, 0.948]	Good to excellent
Vitamin C, mg/day	0.810 [0.642, 0.893]	Moderate to good
Food groups		
Fruits, g/day	0.848 [0.766, 0.902]	Good
Vegetables, g/day	0.837 [0.749, 0.895]	Moderate to good
Cereals and grains, g/day	0.813 [0.705, 0.882]	Moderate to good
Legumes, g/day	0.914 [0.866,0.945]	Good to excellent
Tubers, g/day	0.876 [0.796, 0.923]	Good to excellent
Milk and dairy products, g/day	0.796 [0.691, 0.868]	Moderate to good
Meats, g/day	0.826 [0.735, 0.888]	Moderate to good
Eggs, g/day	0.771 [0.656, 0.851]	Moderate to good
Fish, g/day	0.956 [0.930, 0.972]	Excellent
Oils and fats, g/day	0.383 [0.166, 0.565]	Poor to moderate
Non-alcoholic drinks, g/day	0.760 [0.641, 0.843]	Moderate to good
Meal timing		
Breakfast, hh:mm	0.998 [0.997, 0.999]	Excellent
Mid-morning snack, hh:mm	1.000 [1.000, 1.000]	Excellent
Lunch, hh:mm	1.000 [1.000, 1.000]	Excellent
Mid-afternoon snack, hh:mm	0.902 [0.836, 0.942]	Good to excellent
Dinner, hh:mm	0.932 [0.888, 0.959]	Good to excellent

TEI, total energy intake. CI, confidence interval. ^1^ ICC outcomes: excellent: >0.90; good: >0.75–0.90; moderate: 0.50–0.75; poor: <0.50.

## Supplementary Materials

The following supporting information can be downloaded at https://www.mdpi.com/article/10.3390/nu15081824/s1, Table S1. Mean values of dietary intake and meal timing estimated through the test (3-day image based food record–Remind app) and reference (3-day handwritten food record) methods. Figure S1. Bland-Altman plots showing mean difference vs. mean intakes (solid line) between the test (Remind app) and reference (3-day handwritten food record) methods, and the lower and upper limits of agreement (dotted lines) for food group intake.
