# Supplementary material for: Relative Validity and Reliability of the Remind App as an Image-Based Method to Assess Dietary Intake and Meal Timing in Young Adults
Source: Nutrients. 2023 Apr 10;15(8):1824. doi: 10.3390/nu15081824 (PMC10146256; doi:10.3390/nu15081824)

Table S1. Mean values of dietary intake and meal timing estimated through the test (3-day image based food record – Remind app) and reference (3-day handwritten food record) methods.

Dietary intake	Test method intake (n=71)	Reference method intake (n=71)
Energy, kcal/day	1470.1 (303.6)	1501.1 (332.2)
Macronutrients		
Carbohydrate		
g/day	154.8 (34.0)	160.9 (38.1)
%TEI	42.4 (5.9)	43.1 (5.2)
Protein		
g/day	78.1 (18.3)	78.7 (20.2)
%TEI	21.5 (3.8)	21.2 (3.9)
Fat		
g/day	59.3 (17.6)	59.6 (18.6)
%TEI	35.8 (6.1)	35.3 (5.9)
Saturated fat, g/day	18.5 (6.8)	18.5 (6.6)
Monounsaturated fat, g/day	22.2 (7.6)	23.1 (9.2)
Polyunsaturated fat, g/day	10.2 (3.9)	10.2 (3.9)
Cholesterol, mg/day	248.3 (110.2)	245.3 (116.8)
Dietary fiber, g/day	21.5 (7.7)	21.8 (8.0)
Micronutrients		
Calcium, mg/day	616.4 (227.0)	646.2 (228.1)
Iron, mg/day	9.9 (2.7)	10.4 (3.1)
Magnesium, mg/day	261.8 (72.5)	268.0 (79.7)
Phosphorus, mg/day	1036.2 (283.6)	1075.8 (302.5)
Potassium, mg/day	2526.0 (704.1)	2700.6 (773.7)
Zinc, mg/day	7.3 (1.9)	7.7 (2.2)
Vitamin A, µg/day	728.1 [481.1 – 1004.8]	726.8 [492.1 – 1114.9]
Vitamin D, µg/day	1.8 [1.0 – 3.6]	2.1 [1.1 – 3.9]
Vitamin E, mg/day	7.4 (2.7)	7.9 (3.2)
Vitamin B1, mg/day	1.1 (0.3)	1.1 (0.4)
Vitamin B2, mg/day	1.2 (0.4)	1.2 (0.4)
Vitamin B3, mg/day	16.9 (6.1)	18.0 (6.0)
Vitamin B6, mg/day	1.7 (0.5)	1.8 (0.5)
Folates, µg/day	243.5 (94.1)	260.4 (100.9)
Vitamin B12, µg/day	2.9 [2.1 – 4.5]	2.9 [2.1 – 4.9]
Vitamin C, mg/day	100.3 (50.0)	116.4 (62.0)
Food groups		
Fruits, g/day	167.0 [107.7 – 233.7]	164.0 [113.3 – 246.7]
Vegetables, g/day	179.7 [100.0 – 262.3]	190.0 [95.0 – 287.0]
Cereals and grains, g/day	183.7 (66.4)	171.5 (61.1)
Legumes, g/day	47.7 [0.0 – 75.0]	36.0 [0.0 – 75.0]
Tubers, g/day	41.7 [0.0 – 87.7]	50.0 [0.0 – 116.7]
Milk and dairy products, g/day	211.1 (103.9)	224.7 (101.5)
Meats, g/day	93.3 [38.3 – 140.0]	103.3 [38.3 – 143.3]
Eggs, g/day	18.3 [0.0 – 36.7]	18.3 [0.0 – 36.7]
Fish, g/day	20.0 [0.0 – 58.3]	26.7 [0.0 – 55.0]
Oils and fats, g/day	9.0 [6.0 – 13.0]	7.7 [4.7 – 13.7]
Non-alcoholic drinks, g/day	61.3 [6.0 – 139.0]	45.0 [0.0 – 116.7]
Meal timing		
Breakfast, hh:mm	09:28 (01:00)	09:27 (01:00)
Mid-morning snack, hh:mm	11:25 (01:04)	11:22 (01:05)

Lunch, hh:mm	14:10 (00:34)	14:11 (00:34)
Mid-afternoon snack, hh:mm	17:58 (00:54)	17:57 (01:00)
Dinner hh:mm	21:31 (00:34)	21:31 (00:37)

TEI, Total energy intake. Values are expressed as means ± standard deviations for normally distributed variables and median and interquartile range for non-normally distributed variables.

Figure S1. Bland-Altman plots showing mean difference vs. mean intakes (solid line) between the test (Remind app) and reference (3-day handwritten food record) methods, and the lower and upper limits of agreement (dotted lines) for food group intake.

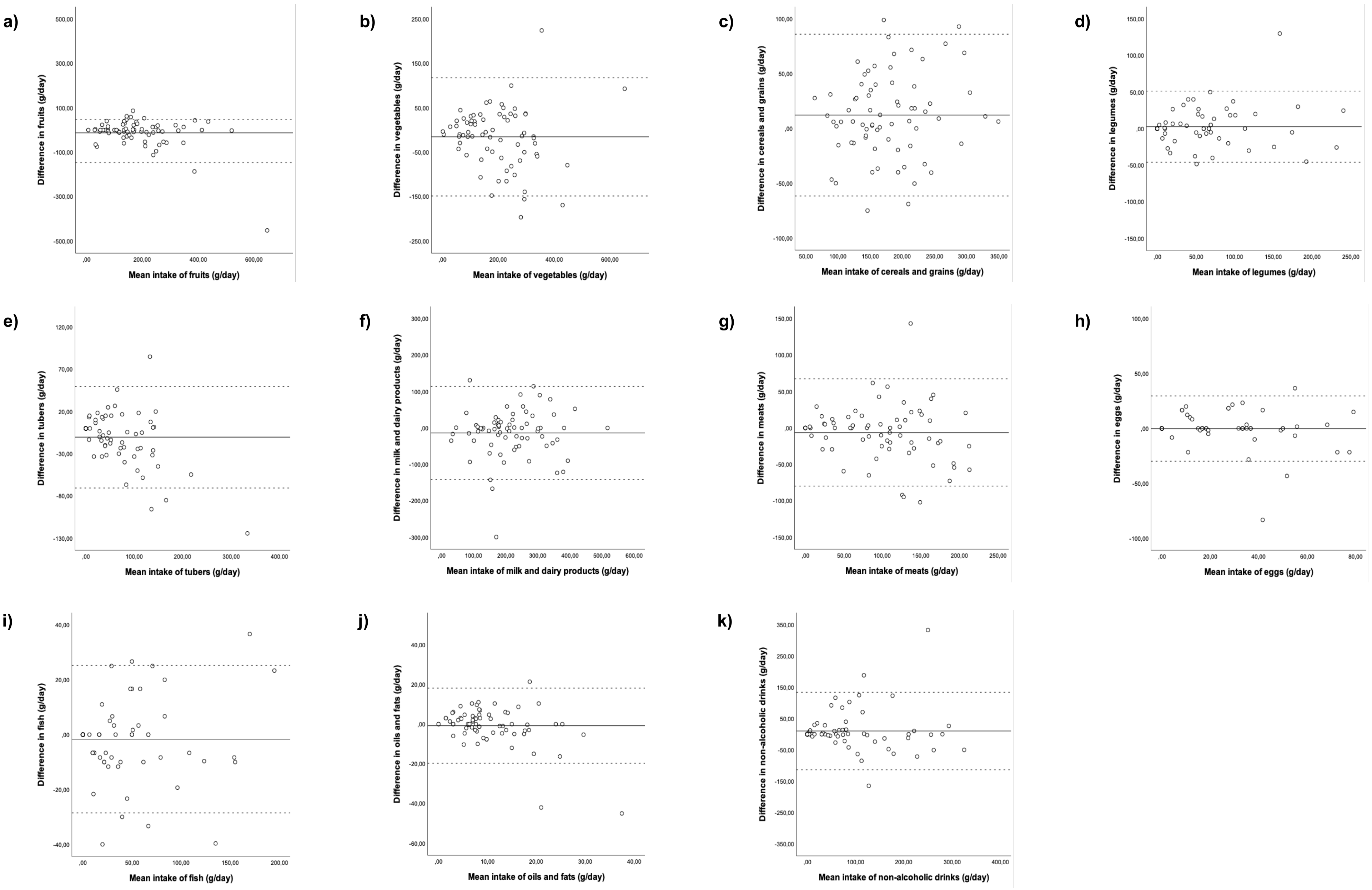

Supplement: Supplementary file 1 [file nutrients-15-01824-s001.zip › nutrients-2318263-supplementary.pdf]
